# Basilar artery thrombosis and Wallenberg syndrome in a patient with uncontrolled hypertension

**DOI:** 10.1016/j.radcr.2024.03.043

**Published:** 2024-05-06

**Authors:** Nazim Dakaj, Kaltrina Gocaj, Serbeze Kabashi, Kreshnike Dedushi, Vullnet Blakaj, Alba Goçaj

**Affiliations:** a Assistant Professor at Alma Mater Europea” Rezonanca”, Specialist of Neurology, KSHM, Rezonanca, Veterrnik 10000, Prishtine, Kosove; b Radiology Resident in University Clincial Center of Kosova, Rrahim Beqiri, Pika Exclusive Llam B-41, Prishtine, Kosove; c Professor Dr. at the University of Prishtina, Radiologist at University Clinical Center of Kosova, KSHM, Rezonanca, Veterrnik 10000, Prishtine, Kosove; d Professor Asisstant at the University of Prishtina, Radiologist at University Clinical Center of Kosova, Swiss Village, Cagllavice, pn, Kosove; e Radiology Resident at the University Clinical Center of Kosova, UCCK, Lagja e Spitalit, 10000, Prishtine, Kosove; f Medical Doctor at Primary Care Urgency Center of Prizren, Rr.Jonuz Krasniqi, pn, 20000 Prizren, Kosove

**Keywords:** Cerebellar signs, Basilar dissection, Lateral medullary syndrome, Stroke, Hypertension

## Abstract

Presented here is a compelling case of a patient with a history of untreated hypertension, highlighting symptoms indicative of Wallenberg syndrome, including acute-onset dizziness, visual disturbances, continuous vomiting, difficulty walking, and an altered level of consciousness. This case's significance lies in its clinical presentation and in the diagnostic journey undertaken to elucidate its underlying pathology.

Throughout the patient's hospitalization, a comprehensive assessment incorporating clinical, laboratory, and imaging techniques was conducted to delineate the extent of their condition. Of particular significance were the findings derived from MRI and MRA examinations of the endocranium, which provided crucial insights into the underlying pathophysiology.

The MRI revealed multifocal ischemic lesions, pointing towards basilar artery thrombosis affecting both vertebral branches and displaying characteristic features associated with Wallenberg syndrome. Notably, the patient's lack of antihypertensive, anticoagulant, or antiplatelet therapy underscores the importance of addressing modifiable risk factors early in the disease course.

This case serves as a poignant reminder of the complexities inherent in cerebrovascular diseases, highlighting the imperative of prompt recognition and management of predisposing factors. By presenting this case, we aim to underscore the clinical significance of timely intervention in mitigating potential complications of hypertension, such as cerebral artery thrombosis, thereby emphasizing the importance of proactive patient care and risk factor modification in clinical practice.

## Introduction

The intricate network of the vertebral and basilar arteries forms a vital conduit for supplying blood to a myriad of vessels within the posterior circulation. Occlusions within these arteries are implicated in approximately one-fifth of all strokes. The clinical spectrum of basilar artery obstruction spans from mild, transient symptoms to severe, and debilitating strokes.

Conversely, Wallenberg Syndrome, also recognized as Lateral Medullary syndrome, represents a neurological disorder characterized by symptoms arising from ischemia or infarction of the lateral aspect of the medulla oblongata. Coined after Adolf Wallenberg, a distinguished neurologist and neuroanatomist, this syndrome predominantly arises from occlusion of the vertebral artery or the posterior inferior cerebellar artery (PICA). These arteries supply critical regions of the medulla oblongata, including the choroid plexus, Tela choroidea of the fourth ventricle, tonsils, inferior vermis, and inferior portions of the cerebellum. Various etiological factors, such as arterial dissection, atherosclerosis, embolism, or thrombosis, contribute to the impediment of blood perfusion.

The identification of Wallenberg syndrome in emergency settings is paramount, particularly due to its association with lateral medullary infarction and vertebral artery dissection. A positive prognosis for recovery from lateral medullary syndrome is anticipated with optimized therapeutic approaches.

Historically, the understanding of clinical aspects related to posterior circulation ischemia has progressed at a slower pace compared to anterior circulation ischemia. Preceding the mid-1980s, catheter angiography and computed tomography (CT) were indispensable for visualizing brain and vascular structures within the posterior circulation. However, the accurate delineation of brain lesions remained elusive until the advent of magnetic resonance imaging (MRI). The emergence of MRI posed challenges in swiftly and accurately identifying the vascular lesions responsible for the condition, thereby impeding effective therapeutic interventions.

## Case presentation

Patient Information: A 50-year-old male presented with a constellation of symptoms suggestive of neurological dysfunction. The patient reported an acute onset of dizziness, accompanied by visual disturbances, difficulty walking, and continuous vomiting.

His symptoms emerged acutely a few days prior to his initial hospitalization, with subsequent deterioration and disturbed consciousness during his hospital stay.

The patient had a history of hypertension but was not currently receiving antihypertensive, anticoagulant, or antiplatelet therapy.

Upon examination, the patient appeared somnolent but maintained normal vital signs. His physical examination revealed a range of neurological deficits, including reduced muscle tone and reflexes, weakness in the upper and lower limbs, positive Babinski sign, and dysarthric speech. Additionally, he displayed singultus (hiccup), suggestive of cranial nerve involvement.

A Glasgow Coma Scale (GCS) score of 13 points (E3, V4, M6) indicated mild impairment of consciousness. Neurological assessments revealed bilateral normal nutrition in the upper and lower limbs, albeit with reduced muscle tone, and reflexes on the right side. Weakness was noted in the right upper and lower limb muscles, without involuntary movements. The patient exhibited rapid left-beating nystagmus, preserved facial muscle response, and symmetric bulbar functions. However, dysmetria in both hands, especially on the right side, was evident during cerebellar testing.

Overall, the patient presented with a range of neurological deficits consistent with a complex neurological disorder.

This case highlights the importance of a comprehensive clinical evaluation and neurological examination in patients presenting with acute neurological symptoms. Further investigation, including neuroimaging studies, was warranted to elucidate the underlying etiology of his condition.

In response to the patient's clinical complaints, diagnostic imaging studies were conducted, with MRI of the endocranium playing a pivotal role. Specifically, the MRI revealed an ischemic lesion indicative of posterior circulation involvement, attributed to basilar artery thrombosis affecting the vertebral artery and posterior inferior cerebellar artery (PICA) territories. These imaging modalities provided intricate anatomical and functional details crucial for precise diagnosis and subsequent management of the patient's condition. The images were acquired using a 1.5 Tesla MRI scanner, employing standard imaging sequences including T1-weighted, T2-weighted, FLAIR (fluid-attenuated inversion recovery), and DWI (diffusion-weighted imaging) sequences.

**Fig. 1:**
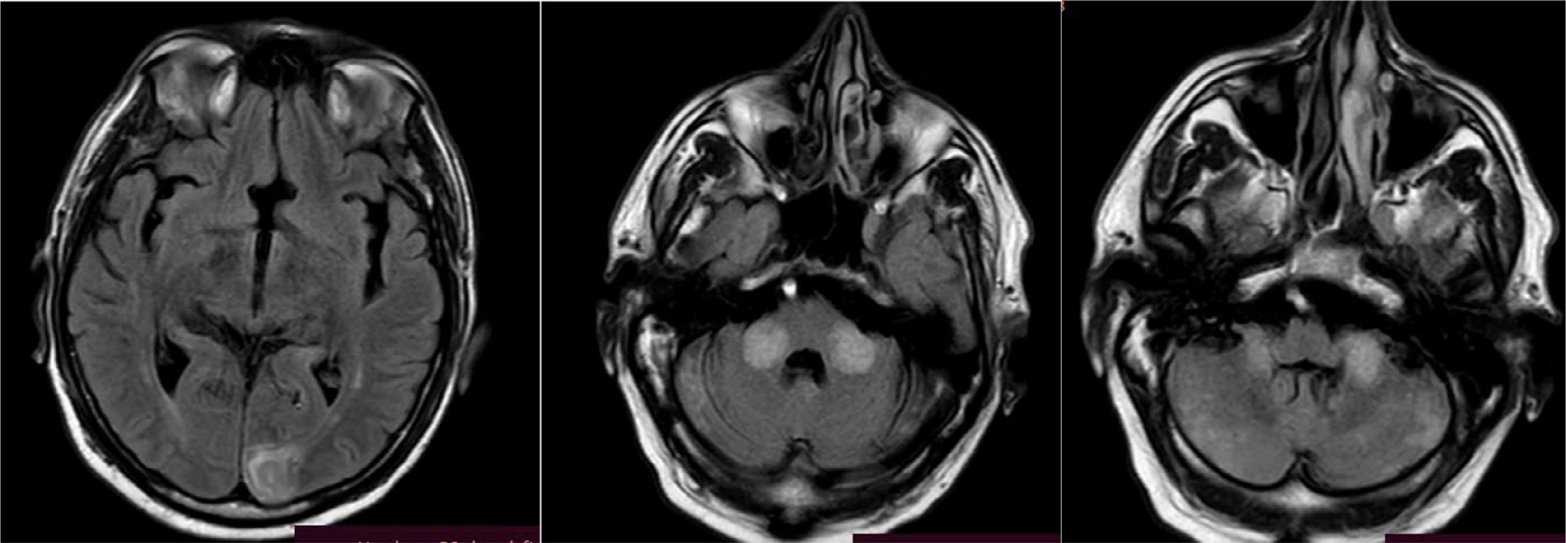
Presents an axial FLAIR sequence MRI of the cranial cavity, highlighting hyperintensities observed in the left occipital lobe.Figs. 2 and 3 Depict bilateral multifocal signal anomalies localized to the infratentorial region, as visualized through the FLAIR sequence.

**Figs. 4, 5, and 6:**
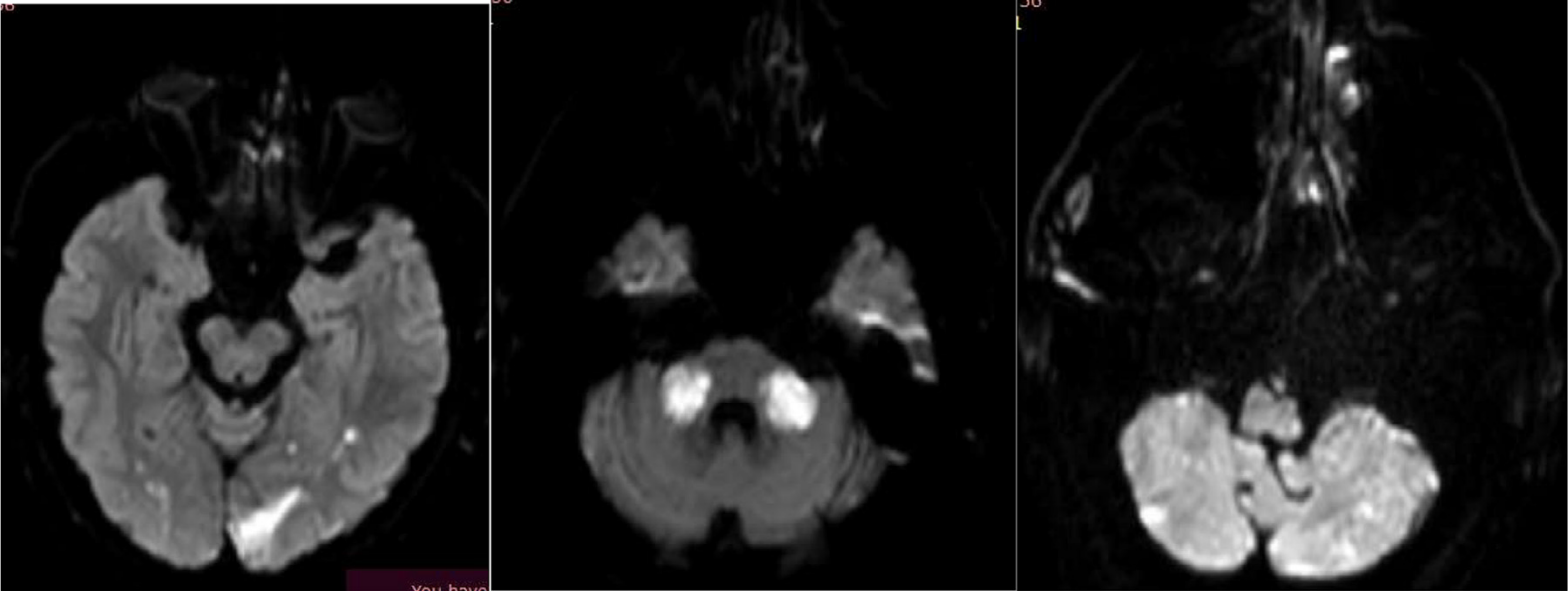
Demonstrate diffusion-weighted imaging (DWI) sequences revealing restricted water diffusion, indicative of ischemic lesions in the vertebrobasilar and posterior inferior cerebellar artery (PICA) regions.

**Figs. 7, 8, and 9:**
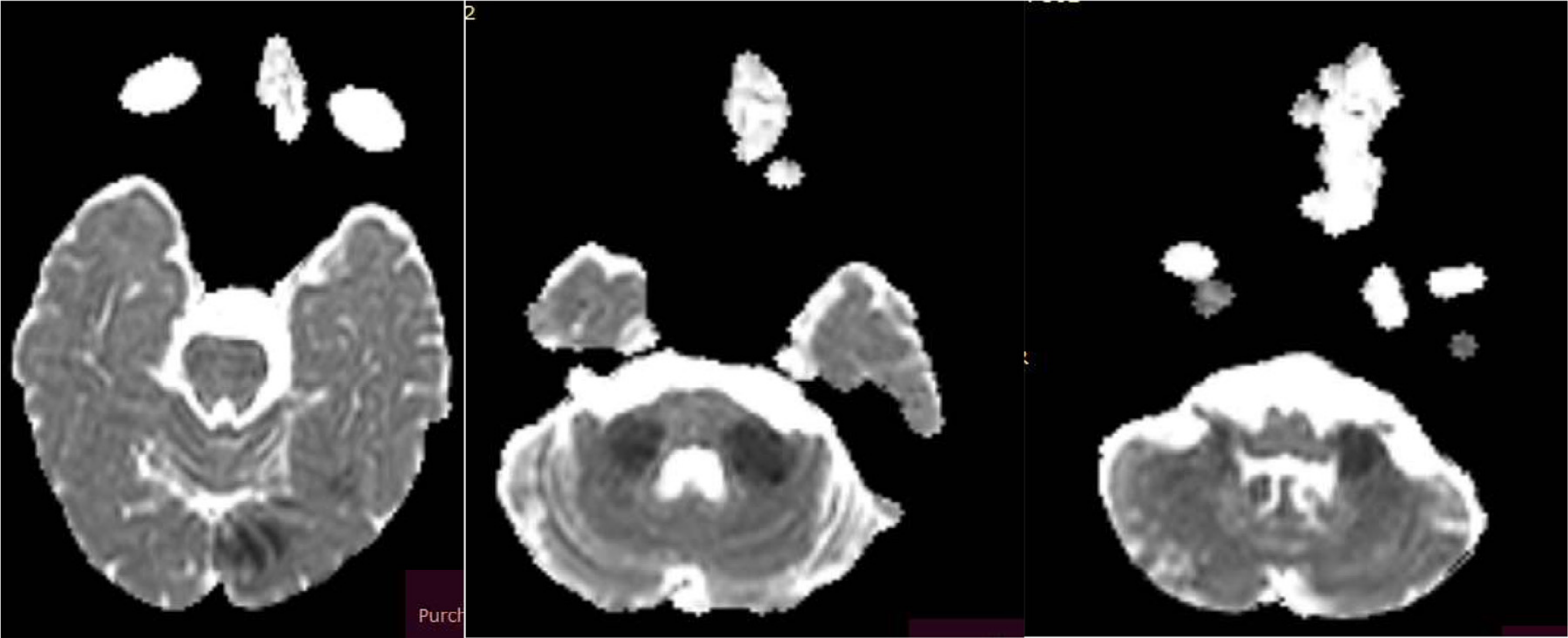
The axial apparent diffusion coefficient (ADC) images validate the presence of genuine water diffusion restriction within the aforementioned zones.

**Fig. 10:**
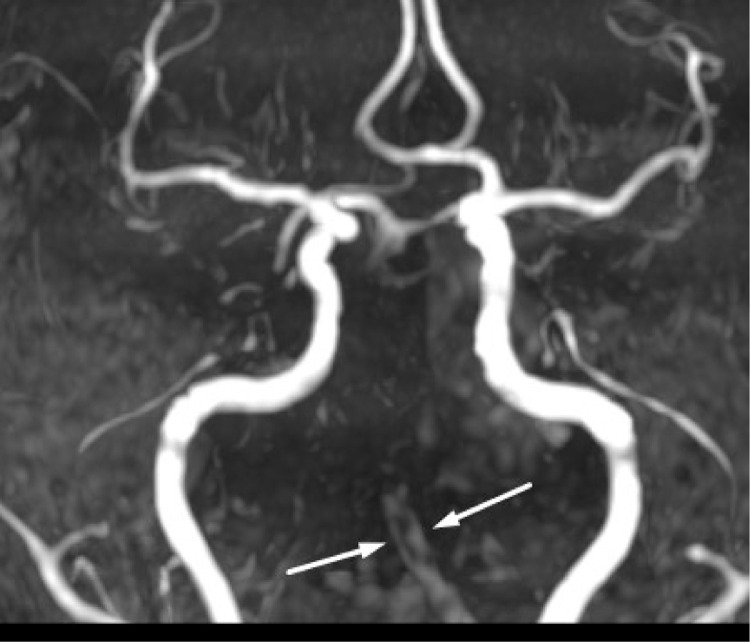
TOF (Time-of-Flight) images from magnetic resonance angiography (MRA) illustrate the basilar artery, depicting both dissection and thrombosis.

The diagnostic process for stroke utilizing MRI involves a multifaceted approach to delineate the underlying pathology. Initially, hyperintensities observed on FLAIR (Figs. 1–3) sequences across various supra and infratentorial brain regions serve as indicators of possible ischemic events. Subsequently, DWI sequences (Figs. 4–6) are employed to assess water diffusion, with restricted diffusion patterns indicative of ischemic tissue. Confirmation of restricted diffusion is corroborated through ADC mapping (Fig. 7–9). Furthermore, MRA (Fig. 10) elucidates vascular abnormalities, such as opacification patterns suggestive of basilar artery thrombosis or dissection.

## Discussion

Wallenberg syndrome, which is also known as lateral medullary syndrome, results from an infarct happening in the region supplied by the posterior inferior cerebellar artery. The condition is manifested with a range of symptoms such as dizziness, uncontrolled eye movements, double vision, and features of Horner's syndrome, besides that other symptoms include facial redness on the effected side, dry skin, voice changes, difficulty swallowing and speaking, loss of gag reflex, imbalance, altered taste, facial discomfort and tingling, and reduced blink reflex. Understanding neuroanatomy is crucial for diagnosing this condition [1]. LMS is an uncommon type of stroke affecting the brainstem, and it has a positive outlook when promptly hospitalized and treated. The most effective diagnostic tool for identifying LMS is brain MRI, particularly the diffusion sequence [2]. Alongside the other clinical conditions our patient experienced, persisting hiccups, or singultus, that at first sight are of no significant medical concern, however, persistent hiccups lasting over 48 hours (about 2 days), as shown in this case, can warrant more comprehensive medical investigations. The underlying physiological and neuroanatomical mechanisms for hiccups are not entirely clear, though they are presumed to involve parts of the brain stem, the respiratory centers, and the phrenic nerve nuclei. With the medulla oblongata playing such a critical role in hiccup reflex arc, it is not surprising that disruptions or damages to this region, like in the case of LMS, can lead to persistent hiccups [3]. Hypertension stands as the underlying cause for the occurrence of the stroke in The patient under consideration, emphasizing the critical role of controlling and managing blood pressure to prevent such serious vascular events. Uncontrolled hypertension amplifies the susceptibility to heart attack, heart failure, kidney disease, stroke, and cognitive decline [[1], [4], [5]]. Suboptimal control rates are, in part, ascribed to a deficiency in hypertension awareness. Improving this awareness holds promise for advancing control rates [6]. In accordance with the metanalysis of findings Upoyo et al., 80.73% of individuals who experienced a stroke had uncontrolled blood pressure, and 75.11% exhibited nonadherence to antihypertensive medication. The primary causes of treatment nonadherence included forgetfulness (58.08%), a lack of confidence in maintaining long-term antihypertensive treatment (27.75%), and a failure to recognize the significance of prolonged treatment (24.75%) [7].

## Conclusion

This case report underscores the multifaceted presentation of Wallenberg syndrome, particularly in a patient with a history of uncontrolled hypertension, accentuating the significance of early diagnosis and intervention. The absence of antihypertensive, anticoagulant, or antiplatelet therapy in this patient's medical history further emphasizes the crucial role of managing modifiable risk factors in the prevention of severe complications such as lateral medullary infarction. The clinical intricacies and potential long-term complications arising from Wallenberg syndrome necessitate thorough understanding, prompt intervention, and continuous research to improve patient outcomes and quality of life.

## Disclosures

The authors do not have any financial disclosures.

## Declaration of generative AI and AI-assisted technologies in the writing process

During the preparation of this work the authors used BERT (Bidirectional Encoder Representations from Transformers) in order to draft sections of scientific manuscript. After using this tool/service, the author(s) reviewed and edited the content as needed and takes full responsibility for the content of the publication.

## Legend

Diagnostic imaging was performed at the University Clinical Center of Kosovo, where images were acquired using a 1.5 Tesla MRI scanner. Standard imaging sequences, including T1-weighted, T2-weighted, FLAIR (fluid-attenuated inversion recovery), and DWI (diffusion-weighted imaging) sequences, were employed to capture comprehensive anatomical and functional information.

## Patient consent

Written informed consent for the publication of this case report was obtained from the patient.

## References

[1] Miao H-L, Zhang D-Y, Wang T, Jiao X-T, Jiao L-Q (2020). Clinical importance of the posterior inferior cerebellar artery: a review of the literature. Int J Med Sci.

[2] Ahmed Ibrahim A, Bakir A, Osman Sidow N, Mohamed Ali A, Farah Osman M, Ahmed A (2023). Lateral medullary syndrome: uncommon form of brainstem stroke. Ann Med Surg (Lond).

[3] Gasca-González OO, Pérez-Cruz JC, Baldoncini M, Macías-Duvignau MA, Delgado-Reyes L. (2020). Neuroanatomical basis of Wallenberg syndrome. Cir Cir.

[4] Mattle HP, Arnold M, Lindsberg PJ, Schonewille WJ, Schroth G (2011). Basilar artery occlusion. Lancet Neurol.

[5] Saleem F, M Das J. (2022). StatPearls.

[6] Ahuja R, Ayala C, Tong X, Wall HK, Fang J. (2018). Public awareness of health-related risks from uncontrolled hypertension. Prev Chronic Dis.

[7] Setyo Upoyo A, Setyopranoto I, Suseani Pangastuti H (2021). The modifiable risk factors of uncontrolled hypertension in stroke: a systematic review and meta-analysis. Stroke Res Treatm.

